# Community Flux Balance Analysis for Microbial Consortia at Balanced Growth

**DOI:** 10.1371/journal.pone.0064567

**Published:** 2013-05-31

**Authors:** Ruchir A. Khandelwal, Brett G. Olivier, Wilfred F. M. Röling, Bas Teusink, Frank J. Bruggeman

**Affiliations:** 1 Molecular Cell Physiology, Faculty of Earth and Life Sciences, VU University Amsterdam, Amsterdam, The Netherlands; 2 Systems Bioinformatics, Faculty of Earth and Life Sciences, VU University Amsterdam, Amsterdam, The Netherlands; 3 Netherlands Institute for Systems Biology (NISB), Amsterdam, The Netherlands; University of Rostock, Germany

## Abstract

A central focus in studies of microbial communities is the elucidation of the relationships between genotype, phenotype, and dynamic community structure. Here, we present a new computational method called community flux balance analysis (cFBA) to study the metabolic behavior of microbial communities. cFBA integrates the comprehensive metabolic capacities of individual microorganisms in terms of (genome-scale) stoichiometric models of metabolism, and the metabolic interactions between species in the community and abiotic processes. In addition, cFBA considers constraints deriving from reaction stoichiometry, reaction thermodynamics, and the ecosystem. cFBA predicts for communities at balanced growth the maximal community growth rate, the required rates of metabolic reactions within and between microbes and the relative species abundances. In order to predict species abundances and metabolic activities at the optimal community growth rate, a nonlinear optimization problem needs to be solved. We outline the methodology of cFBA and illustrate the approach with two examples of microbial communities. These examples illustrate two useful applications of cFBA. Firstly, cFBA can be used to study how specific biochemical limitations in reaction capacities cause different types of metabolic limitations that microbial consortia can encounter. *In silico* variations of those maximal capacities allow for a global view of the consortium responses to various metabolic and environmental constraints. Secondly, cFBA is very useful for comparing the performance of different metabolic cross-feeding strategies to either find one that agrees with experimental data or one that is most efficient for the community of microorganisms.

## Introduction

In nature, microbes generally occur in communities. These microbial communities play important roles: they are essential for global nitrogen, carbon and energy cycling [1] and contribute to a healthy human physiology as part of our oral and gut flora [2]. In such complex systems, the physiology, behavior, and fitness of the species are interdependent. It is a major challenge to understand how the interplay between microbes determines community dynamics and robustness, and how the genotype of each of the microorganisms ultimately influences ecosystem properties.

Today, advanced molecular methods (meta-omics) facilitate the detailed characterization of microbial communities, providing information at an unprecedented level of molecular detail. These methods catalogue the active molecular processes, the ecotypes present, and report the identity and abundances of specific microbial species [3]. While such approaches are generally high-throughput, comprehensive and broadly applicable, they give little insight into the rationales behind the metabolic behaviors of individual microbial species. Why do microbes choose a particular physiological state out of their full range of metabolic capacities? How do these decisions depend on the metabolic coupling between species? Which metabolic interactions determine community structure and how do selective pressures influence this? Answering these questions will require integrative computational approaches that link genes to species metabolisms and community-level structure and offer a consistent framework for describing community level interactions [4], [5]. The promise of these methods, combined with in depth molecular characterization, is the rational design, manipulation and control of microbial communities in biotechnology and medicine.

Constraint-based stoichiometric modeling of genome-scale metabolic networks is a set of computational methods developed in systems biology for studying the comprehensive metabolic capacities of organisms [6], [7]. This collection of computational methods considers the entire metabolic network of an organism as reconstructed from genomic and physiological information [8]. Flux distributions in metabolic networks for optimal biomass or product formation can be predicted from the resulting genome-scale stoichiometric models with flux balance analysis (FBA), for instance as function of the nutrient conditions and as a response to enzyme knock-outs [6]. These models generally compute steady states of metabolic networks and consider only reaction stoichiometry and omit enzyme kinetic information [9]. Constraint-based stoichiometric modeling of genome-scale metabolic networks is widely used in biotechnology and medicine [7].

In microbial communities, a new level of complexity is added on top of microbial metabolism that complicates the application of constraint-based stoichiometric modeling to microbial communities. Besides the presence of all metabolic reactions in each of the microorganisms, the exchange of metabolites between species and biomass abundances of each of the microbial species has to be considered. In addition, each of these microorganisms has specific nutrient requirements for growth, which it can meet through metabolic cross-feeding, nutrient-competition or by uptake from the environment. On top of that, selective pressures at the level of single species change the metabolic interactions between species through mutations, which leads to accumulation of genetic variants and co-evolution of metabolic partnerships. These forces together shape the structure of microbial communities. In such systems, the actions of individual species are constrained by their own biochemical processes and by their interactions with other species. Computational methods are essential to address those complex aspects of biological systems [10].

Considerable effort has been invested in recent years to develop suitable computational approaches that, in principle, can consider all the metabolic reactions occurring in a microbial community [11], [12], [13], [14], [15], [16], [17], [18], [19], [20]. These studies differ in the computational and mathematical methods employed, some are for instance limited to mutualistic metabolic interactions or compartmentalized approaches. Compartmentalized approaches [12], [16], [21], [11], [22] to microbial communities consider only metabolite exchanges without the explicit consideration of biomass abundances of individual species, even though this aspect of microbial community composition is a major research subject in microbial ecology. More advanced methods can consider competition for resources and variable biomass abundance. They typically make use of dynamic flux balance analysis [23], [24]. The recently introduced method ‘OptCom’ [20] is arguably the most advanced method and uses sophisticated multi-objective optimization techniques to predict the biomass composition of the community along with the growth rates of each species. OptCom takes a multi-objective optimization (Pareto optimization) approach to interrelate the objectives of individual organisms.

The reason why constraint-based stoichiometric modeling of microbial communities is much more complicated than for single organisms originates from the interdependencies between the metabolic objectives of microorganisms in the community. Generally, in constraint-based stoichiometric modeling, a single metabolic objective is postulated for the organism, such as optimization of biomass yield, to give rise to a manageable solution space of flux distributions [25]. In a microbial community, the metabolic performance (fitness) of each organism is dependent on all others, either directly or indirectly. As a consequence, the community metabolic state in a constraint-based stoichiometric model of the microbial community would have to emerge from the multi-objective optimization of each of those performances, taking into account trade-offs and possibly allowing for suboptimal strategies. Hence, a nonlinear multi-objective optimization perspective appears most appropriate and this is indeed the approach taken by OptCom. In this paper, we show that the multi-objective optimization task greatly simplifies when microbial communities are considered that engage in balanced growth. This leads to the formulation of a new approach for constraint-based modeling of microbial communities, which we shall refer to as community FBA (cFBA). It requires fewer assumptions than full-blown multi-objective optimization approaches and is much easier to interpret.

Balanced growth occurs when internal metabolism is at steady state while the cells grow exponentially at a fixed growth rate. Here, we extend this definition to microbial ecosystems and, hence, require all metabolites (intra- and extracellular) to attain a steady state level. Under those growth conditions, our computational method, community flux balance analysis (cFBA), predicts the fractional biomass abundances of all the participating microorganisms in the community as well as the intra- and extracellular flux distributions and metabolic exchanges. cFBA predicts the complete state of the microbial community engaging in balanced growth and postulates only a single objective. cFBA applies to microbial ecosystems that function in a fairly constant environment, such as specific microbial communities involved in bioremediation, waste water treatment or in laboratory settings, e.g. chemostats.

The community flux balance analysis (cFBA) that we will present in this paper is a direct translation of FBA for single organisms to microbial communities and requires only a few community-specific constraints. It is based on the concept of balanced growth of microorganisms and the corresponding metabolic network states. It is therefore a fundamental description of the microbial community structure resulting from basic concepts of microbiology. It directly applies to the study of stable microbial communities under laboratory conditions in controlled bioreactors, either resulting from laboratory evolutionary experiments or from direct samples from the environment, or for microbial communities in nature that are exposed to prolonged stable environmental conditions. cFBA predicts the optimal flux distribution, growth rate, and abundance of all species in the consortium as well as the exchange fluxes between species and the community environment. As a proof of concept, we first investigate a simple microbial community in which species are mutually dependent for their growth. Next, we study an evolved syntrophic *E. coli* consortium with genome-scale models.

## Results

### A Methodology for Constraint-based Stoichiometric Modeling of Metabolism of Microbial Consortia

Constraint-based stoichiometric modeling of a microbial consortia requires the coupling of the metabolic networks of the interacting species. To highlight the essential steps, we illustrate our methodology first by considering interacting microorganisms described by highly simplified stoichiometric models consisting of lumped metabolic processes: anabolism, catabolism, respiration and product formation. In all cases, the reaction networks are balanced with respect to elemental composition, charge and degree of reduction [26]. The application of our methods to genome-scale stoichiometric models is exactly the same as to the small stoichiometric models that will be used in this section.

**Figure 1:**
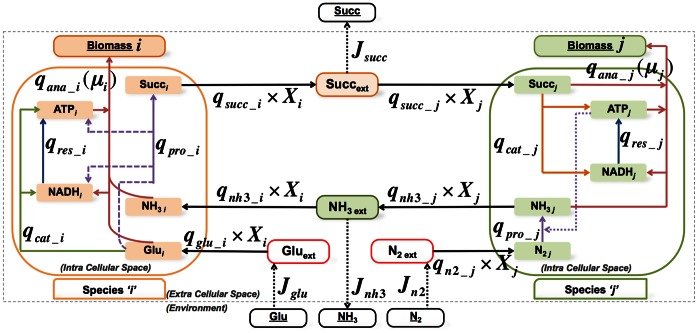
Metabolic network overview of a microbial community of two microorganisms engaging in metabolic cross feeding. Species *i* and *j* exchange ammonium (NH_3_) and succinate (Succ). These metabolites are allowed to overflow into the environment. Species *i* and *j* respectively take up glucose (Glu) and dinitrogen gas (N_2_) supplied by the environment. Each organism operates at an intracellular metabolic steady state and contains four coarse-grained metabolic processes: catabolism, anabolism, product formation and respiration. Detailed information about the stoichiometric description of this community can be found in information S1. Three types of reaction rates occur: specific fluxes (*q*’s; in *mol•(gram biomass) ^−1^•h^−^* ^1^), environment fluxes (***J***’s; in *mol•h^−^* ^1^), and specific growth rates (*μ*; *h^−^* ^1^). *X_i_* and *X_j_* denote the biomass abundances of the two microorganisms in gram biomass.

In Figure 1, two microbial species are shown that engage in metabolic cross feeding. Species 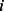 consumes glucose and ammonium and produces succinate. Species 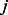 consumes succinate, fixes nitrogen gas and excretes ammonium. When these organisms grow together on glucose and nitrogen supplied by the environment, they engage in an obligatory mutualistic relationship where species 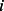 supplies succinate to species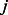 that gives ammonium in return. A stoichiometric description of the metabolism of the entire consortium considers the two species as metabolically coupled. To allow for loose metabolic coupling of these two organisms, overflow of cross-feeding metabolites into the environment is allowed.

Besides the stoichiometric aspects of model merger, we also consider the biomass abundances of each of the participating microorganisms; a feature, which is not explicitly addressed in many existing multi-species FBA approaches, except for OptCom [20]. Several compartmentalized approaches to multi-organism stoichiometric modeling do not explicitly take the biomass abundance into account. As a consequence, the reaction fluxes that these methods predict are net fluxes, which equal the product of the biomass abundance and the specific flux (expressed per unit biomass). Because in many applications specific fluxes and biomass abundances can be independently measured, we aim at considering biomass abundance and specific fluxes separately. Next, we will show that the mass balances of all the variable metabolites in a microbial community can be compactly expressed in terms of the metabolic reaction rates, the microbial biomass abundances, and the community growth rate.

We distinguish three types of reactions: an intracellular, enzyme catalyzed reaction occurring at a specific rate 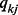 (in *mol•g^−1^•h^−1^*) for reaction 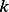 in organism 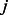, the biomass formation reaction occurring at a specific growth rate 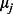 (in *h^−1^*) for organism 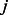, and exchange reactions with the environment denoted by 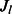 for reaction 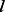, with unit *mol•h^−1^*. The mass balances for the variable metabolites in the community, occurring either intracellularly or extracellularly, will then have the following structure (we omit abiotic conversions between variable metabolites in the environment but those can be added straightforwardly):
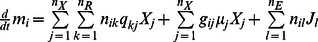
(1)


Here, 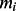 denotes the amount of variable metabolite 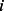. The number of different microbial species, environmental exchange reactions and metabolic reactions equals 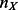, 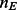 and 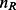, respectively. The stoichiometric coefficient of metabolite 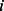 in reaction 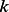 equals 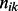; this reaction runs at specific rate 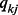 in organism 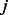 that has a total biomass of 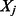 gram. The unit of the stoichiometric coefficient is dimensionless. The resulting dimension of 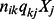 is *mol•h^−1^*. Metabolite 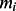 can also be consumed or produced in the biomass formation reaction of an organism; this flux equals 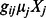. The coefficient 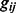 is the stoichiometric coefficient of 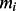 in the biomass reaction of organism 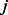 and has as unit 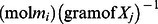. This organism grows at a specific rate 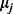 (in *h^−1^*). The resulting unit of 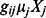 is also *mol•h^−1^*. Finally, metabolite 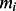 can flow into or out of the environment with rate 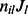 in *mol•h^−1^* with 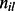 as a dimensionless stoichiometric coefficient.

### A Community at Metabolic Steady State

In our approach, the entire consortium is considered at steady state such that all the mass balances for the metabolites equal zero and the reaction rates remain constant. Equation (1) then indicates that this condition can be maintained if the organism abundances remain fixed and their net growth rate is zero or if the organisms all grow at the same specific growth rate and the exchange rates with the environment increase with the same factor as biomass. The first condition implies that none of the organisms grow but do exchange metabolites; this is a situation that cannot be ruled out in realistic microbial ecosystems. Perhaps, they can persist in periods of dormancy or stasis. We emphasize that in the second scenario, which is the main focus of this paper, the specific growth rate of the community does not have to equal the maximal growth rate of any of its resident microorganisms. The growth rate considered is the one leading to balanced (steady-state) growth.

In that condition, for microorganisms growing at the same specific (exponential) growth rate 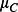 (the community growth rate) from time point 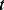 onwards, for any time 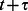, equation (1) modifies to:
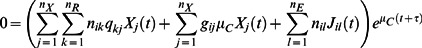
(2)


Here, time 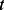 is defined as a time point at which the system achieves a steady state where all metabolite amounts remain fixed. We will allow the community in this state of balanced growth for some finite amount of time, i.e. for 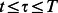 until nutrients run out at 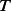. Under this condition, all the mass balances can remain zero at fixed values for the reactions rates and growth rates, provided that the term between brackets remains zero. In this period, the total biomass 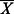 increases exponentially over time as,
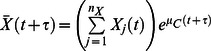



Division of equation (2) with this relation for the total biomass indicates that the biomass fractions 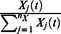 for organism 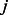, remain fixed over time at their values at time 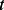,
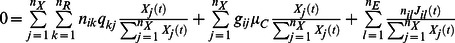



This relationship now holds for the entire period of exponential growth, 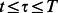; for this duration the biomass fractions, the reaction rates and the growth rate are independent of time. We define the constant biomass fractions as 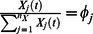 and identify the ratios 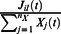 as specific environment exchange fluxes 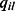 (in *mol•g^−1^•h^−1^*),
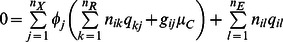
(3)


(Note that 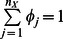). This relationship now holds for all the variable metabolites in the consortium (for all 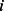) for 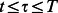. It is an extension of the concept of balanced growth in microbiology for single organisms to microbial communities.

To intuitively understand why unequal specific growth rates would violate the steady-state requirement, a perspective on the mass balances of the exchange metabolites is instructive. If organisms would not grow equally fast, metabolites that are being cross-fed would either deplete or accumulate and the slow growing cross-feeding species would inhibit growth of its fast growing partner. Consider for instance the two species shown in Figure 1; if species 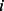 grows faster than 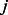 then succinate accumulates – there is not enough 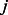 to consume it – and *j* does not make enough ammonium to support species 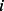 *’*s high growth rate and the growth rate of 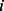 drops. The same applies to species 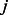 when it grows faster than species 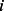. Thus, for a cross-feeding metabolite to be at steady state (in a mutualistic consortium of growing organisms) the producing and consuming organisms need to grow equally fast. Above we have shown that when this condition is met, the fractional biomass remains fixed and the whole consortium can exist in a balanced growth condition.

### Community Flux Balance Analysis Predicts Yields

In community flux balance analysis, equation (3) acts as a constraint that specifies the consequence of balanced growth; this is a hard constraint and cannot be violated. The aim of cFBA is to predict the “variables” from this equation and additional constraints. The variables are: the specific reaction rates (the 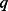
’s), the community growth rate (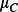) and the fractional biomass abundances (the 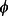
’s). However, fewer balance equations (equal to the number of variable metabolites) will exist than unknown variables (equal to twice the number of microorganisms and the number of reactions) and as a result an infinite set of solutions exists. In such cases of underdetermined problems, one can take a constrained optimization approach. This is indeed the approach taken in flux balance analysis for single organisms: it aims to find a reduced set of solutions that each give rise to the same degree of optimal metabolic functioning, e.g. maximization of ATP or biomass yield. Here, we will take a similar approach for studying microbial community metabolism. We also add more constraints to reduce the solutions to a realistic set; i.e. flux constraints that derive from thermodynamic information about reaction reversibility and measured/estimated upper and lower bounds of specific reaction rates. As a result, this information sets the bounds for every specific reaction rate: 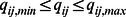.

One approach to predicting the balanced-growth consortium structure, i.e. the flux distributions and fractional biomass abundance, is to search for community structures that optimize the growth rate of the entire consortium. Equation (3) immediately indicates that this problem would be ill posed if none of the fluxes has a maximal bound; because multiplication of the equation with a constant preserves the same solution for the variables, and, hence, the maximal growth rate can become infinite. Here, we follow classical flux balance analysis and solve this problem by imposing a maximal value on one or several nutrient uptake fluxes from the environment. Because of the structure of equation (3), an increase of this upper bound by a certain factor will cause the optimum to increase with the same factor; unless some other flux bound is hit (and then this behavior exists with respect to this new bound). This indicates that the specific growth rate 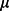 is optimized relative to some specific limiting reaction rate; hence, the ratio of 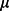 over some 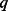 is maximized, which means, in microbiological terms, that the yield of biomass is being optimized and not the growth rate. This indicates that community flux balance analysis predicts optimal biomass yield, as does classical FBA. This state corresponds to the most metabolically efficient consortium state.

While classical flux balance analysis problems are linear in terms of their variables, equation (3) is clearly a nonlinear function of the optimization variables because the specific rates and fractional biomass abundances occur as products. This indicates that the constrained optimization of the community growth rate is not a linear programming problem, which is the usual approach taken in FBA of single organisms. However, for small microbial communities a linear programming based approach remains feasible. By fixing the fractional biomass abundances, the optimization problem becomes linear and a linear programming problem can be formulated to obtain the specific flux values that optimize the community growth rate. Next, the linear program optimization is repeated over the entire range of feasible biomass fractions to identify the vector of biomass fractions at which the community growth rate reaches its global maximum. The linear programming result in this optimal state allows for the identification of the optimal specific flux values. This is the approach that we take in this paper. This approach is explained in more depth in information S1.

### cFBA of an Example Community: Metabolic Limitations of Microbial Consortia

We will analyze the simplified microbial consortium introduced in Figure 1 with cFBA. cFBA suggests that a biomass fraction exists that optimizes the community growth rate. We will show that this state is influenced by the metabolic constraints of the microorganisms and by the nutrient limitations imposed by the characteristics of the environment. These kinds of limitations occur naturally in cross-feeding microbial consortia: species 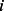 and 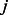 are interdependent and species 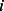 can only grow faster if it delivers more cross-feeding product (i.e. succinate) to 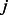 by choosing a more efficient pathway for product formation. However, there will be a limit to the yield of product on the growth substrate. Suppose, species 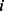 invests a certain amount of resources in product formation (i.e. succinate) to attain a certain growth rate; for species 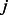 this implies, to attain the same growth rate, it should take up more of succinate (cross-feeding metabolite) and at the same time excrete more of ammonium (its crossing feeding product) to allow species 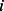 to grow equally fast. This continues until either one of the species hit its maximal excretion or uptake capacity for one of the cross-feeding metabolites or the environmental influx of nutrients become limiting. At the same time, the importance of the biomass ratio can also be rationalized. The objective function of the consortium is to achieve the maximal specific growth rate and the organisms cannot grow infinitely fast, because of the environmental and cross-feeding limitations. As a consequence, the only way to enhance the maximal specific growth rate is by adjusting their biomass fractions. The mathematical formulation of this cFBA is explained in information S1.

**Figure 2:**
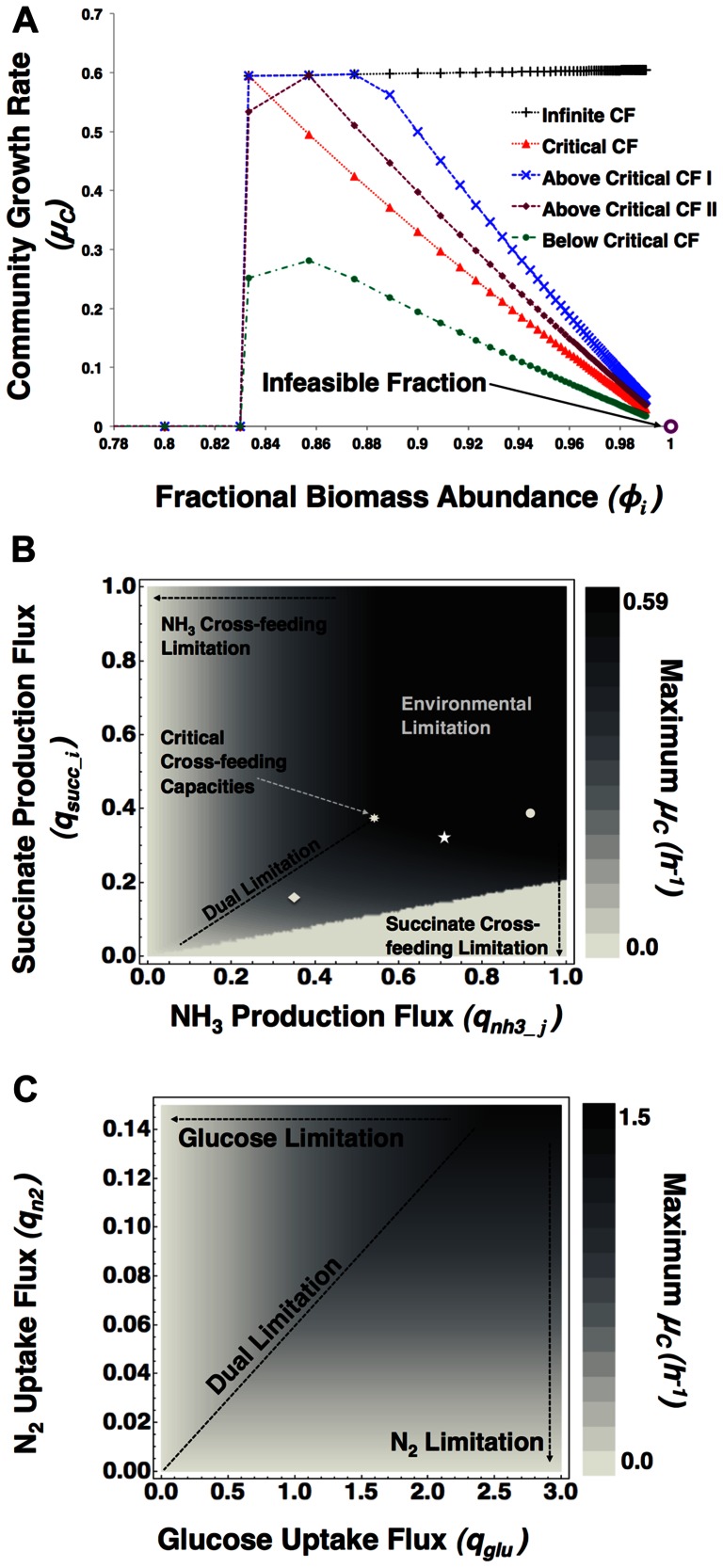
Illustration of environmental and metabolic limitations at the consortium level, for the consortium depicted in Figure 1E. A: Optimal consortium growth rate (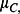), in *h^−1^*, as a function of fractional biomass abundance of species 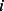 (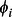) at different cross-feeding (CF) reaction capacities, with all 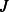
’s in *mol•h^−^* ^1^ and all 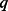
’s in *mol•g^−1^•h^−^* ^1^. We consider a limited glucose (flux: 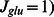 and excess nitrogen (flux: 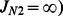 and vary the flux bounds for CF fluxes (i.e. succinate (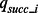) and ammonia (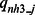) production fluxes) to distinguish different limitation regimes and optimality states: i. infinite CF when, ii. critical CF (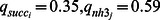), iii. two cases for above critical CF: curve I (for: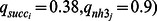 and curve II (for: 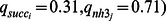, and iv. below critical CF (for: 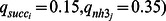. This figure indicates that the CF reactions determine the optimal value of the community growth rate and the optimal fractional biomass abundance. B: A contour plot is generated for the optimal community growth rate 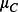 as function of the upper bound of the succinate production flux by species 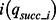 and the ammonia production flux of species 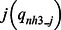. The environmental conditions are the same as in Figure 2A. The different points depict the various cross-feeding regimes distinguished in Figure 2A (• Above Critical CF – I, ★ Above Critical CF – II, ♦Below Critical CF). This figure indicates that the CF fluxes between the organisms determine the optimal community state at a fixed environment. C: Contour plot of the maximum community growth rate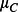, as a function of the environment while cross-feeding capacities are kept unconstrained. This figure indicates that the optimal state of the ecosystem can be determined by specific environmental fluxes.

In Figure 2, we show the results of cFBA for the simplified microbial consortium displayed in Figure 1. We carried out the optimizations in Figure 2 by treating the fractional biomass abundance as a parameter and determine the optimal specific growth rate of the consortium (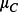) as a function of this parameter. This approach gives an overview of the metabolic behaviors that cross-feeding organisms can display in consortia. In Figure 2A, we explore various limitations that can emerge in microbial consortia. We consider the consortium under the condition that glucose influx from the environment is limiting. When no restrictions apply to the values of cross-feeding reactions, a minimal amount of species 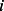 is required to attain a non-zero community growth rate. At low fractional abundance of species 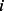, species 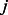 is in high relative abundance and has a demand for succinate to grow. At this condition, the total uptake flux of glucose by species 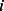
( = 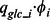) will be low and the production flux of succinate will be too small to sustain the growth rate and amount of species 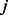 and hence the community cannot be sustained at these fractional biomass abundances. Upon reaching a critical fractional biomass abundance (0.83 and higher for our particular example in Figure 2A) the system can easily sustain growth because the nitrogen gas influx is unlimited, the ammonium production is unlimited, and the succinate requirement decreases progressively. Therefore, eventually the system starts to make succinate or takes up less glucose. Clearly, this is an unrealistic situation: the exchange fluxes of succinate and ammonium and the fixation and assimilation of nitrogen gas are not unlimited but are bounded by the biochemical capacities of the two organisms. Maximal capacities of the cross-feeding reactions can either cause the system to reach a lower maximal community growth rate because the exchange reactions cannot reach high enough rates (‘below critical cross-feeding’ line in Fig. 2A) or cause a reduction in growth rate at low abundances of species 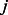 because the ammonium production rate hits it maximal value (‘above critical cross-feeding I & II’ lines in Fig. 2A). Finally, the cross-feeding reaction bounds can be adjusted to specific values to reach a unique maximum for the community growth rate (‘critical cross-feeding’ in Fig. 2A). Clearly, the situation where the cross-feeding reaction bounds are tuned in such a way that a unique fractional biomass abundance achieves maximum growth rate, is an unrealistic scenario. In general, we would expect, and conclude from this analysis, that cFBA predictions of microbial communities lead to a range of fractional biomass abundances that support the maximal growth rate. More cFBA studies of other microbial communities would have to be carried out to study this assertion more carefully.

In Figure 2B, the influence of the cross-feeding reaction bounds on the maximal community growth rate is shown under the environmental conditions of Figure 2A (glucose limitation). At each value for the cross-feeding reaction bounds, community growth rate is maximized and plotted in Figure 2B. A dual limitation line appears in this plot; at this line succinate and ammonium exchange reactions are equally limiting and a reduction of any of them leads to a reduction in community growth rate. Cross-feeding reaction-bound values at the end of this dual-limitation line correspond to the critical values identified in Figure 2A. At any higher values than these critical values, the entire system becomes limited by environmental influx of nutrient, i.e. glucose. In Figure 2C, we study the dependence of the maximal community growth rate on environmental exchange fluxes. While for all the simulations in Figure 2A and 2B glucose intake flux was kept limiting and nitrogen intake flux was in excess, in Figure 2C, the steady-state maximum 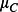 for the consortium is plotted, by limiting glucose or nitrogen intake or both (along dual limitation line) flux values.

These results indicate that knowledge of flux bounds (maximal capacities) is important to assess the types of limitations that microbial consortia encounter and that scanning of those bounds allows for a global view of systemic consortium responses to metabolic and environmental constraints. This scanning of flux bounds is common practice in FBA applied to single species and is normally referred to as phase plane analysis [27]. We show here its relevance and importance for community FBA.

### cFBA to Study a Speciation Event in Long-term Laboratory Evolution of *Escherichia coli*

**Figure 3:**
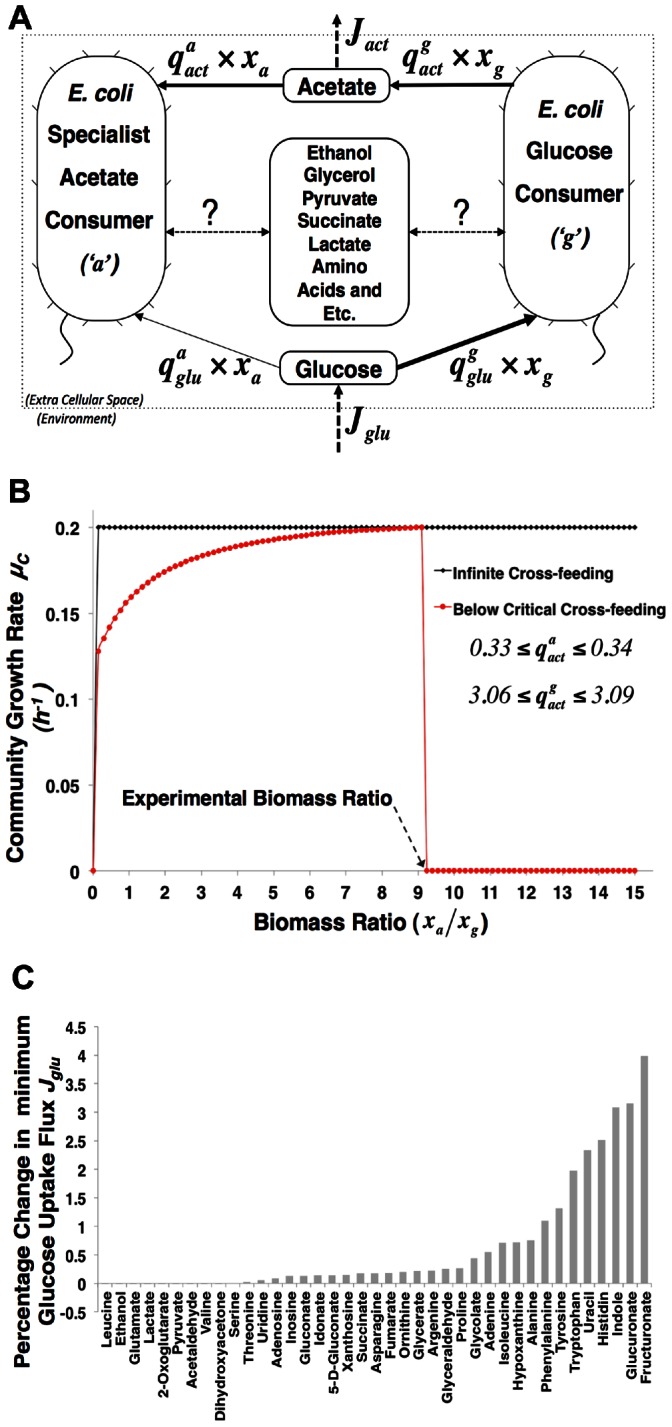
Illustration of cFBA applied to a genome-scale stoichiometric model of a microbial consortium evolved in a chemostat. A: Consortium of two *E. coli* strains; one is a glucose consumer ‘
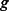
’ and other is a specialist acetate consumer ‘
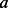
’. They both take up glucose with specific glucose consumption fluxes 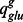
and 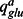 in *mmol•g^−1^•h^−^* ^1^ but ‘
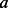
’ does this with lower activity than ‘
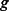
’. Strain ‘
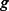
’ produces acetate with flux 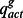 and strain ‘
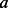
’ consumes it via flux 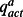. In the chemostat, glucose is provided at a constant rate 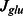. And
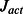
is the acetate production rate (*mol•h^−^* ^1^). Various metabolites could be cross-fed between both organisms besides acetate, which leads to the question whether those alternative metabolites can be predicted by cFBA. B: A biomass ratio scan was performed and the community growth rate 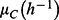
is plotted as a function of the steady-state biomass ratio. The following parameter were determined from the experimental data of Rosenzweig et al. (1994):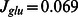; 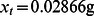; dilution rate (D) = 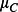 = 0.2; and steady state biomass ratio 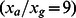. To plot the ‘Below Critical Cross-feeding’ curve, cross-feeding fluxes were constrained as indicated in the plot, while for the ‘Infinite Cross-feeding” curve, unconstrained acetate cross-feeding capacities were assumed. C: Percentage change in minimum glucose uptake rate 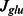
needed to achieve the growth rate 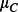 of 0.2 *h^−^* ^1^ for alternative cross-feeding metabolites (one-at-a-time).

Helling & Vargas [28] observed the evolutionary emergence of polymorphisms in an *Escherichia coli* culture that was grown for 765 generations in a glucose-limited continuous culture. In a follow-up experiment, Rosenzweig et al [29] isolated a number of *E. coli* strains from these polymorphic populations. It turned out that one strain had evolved to become a specialized acetate consumer ‘
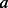
’ and another strain had become the main glucose consumer ‘
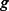
’ producing acetate (Figure 3A). The glucose uptake capacity of strain ‘
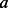
’ proved lower than that of strain ‘
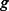
’ when measured in isolated cultures. We mimicked this in our simulations with an extra constraint (
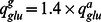
) derived from the same experiment. Values for the steady state biomass ratio and glucose input flux were taken directly from Rosenzweig et al. [29]. Total biomass amount was estimated by translating cell densities into gram dry weight, taking 0.95 picogram as the weight of a single *E. coli* cell [30]. In all the following simulations, the total biomass amount (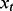) was kept fixed in agreement with the experiment. We used the genome-scale stoichiometric model of *Escherichia coli* K-12 strain [31] for our simulations. We made a stoichiometric model for the acetate consumer and the glucose consumer and studied this system with cFBA.

Instead of parameterizing fractional biomass abundance of one organism of a community (Figure 2A), here we have optimized the consortium growth rate as function of the biomass ratio (Figure 3B). Since, the biomass fraction can be expressed in terms of the biomass ratio the optimization is essentially the same as previously. The biomass ratio scan was performed by either constraining the capacity of the acetate production flux by strain ‘
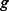
’ or the acetate consumption flux by strain ‘
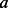
’ (leading to a below critical CF curve as introduced above) or keeping them unconstrained (leading to an infinite CF curve). The “Infinite CF curve” gives us a range of optimal biomass ratios, within which it is theoretically possible for the community to sustain a maximal community growth rate. The experimentally measured biomass ratio 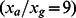 lies within this range; and is most likely set by the biochemical limitations on the capacities of acetate uptake and production in the chemostat.

Next, we asked what the acetate cross-feeding flux values should be to give rise to the measured biomass ratio; the corresponding curve we denote as the below CF curve. Though the acetate cross-feeding fluxes were not measured during the experiment, our approach predicts a probable range of flux values that gives rise to the measured biomass ratio, glucose-uptake rate, and the specific-growth rate of both of the strains. This illustrates how cFBA can be used to predict cross-feeding fluxes in a microbial community using a genome-scale metabolic model of the community.

We also tested whether cFBA predicts alternative cross-feeding metabolites that can explain the experimental data equally well as acetate does, to illustrate one useful application of cFBA. It was concluded in Helling et al. [28] that the newly evolved strain would be an acetate consumer. With cFBA we tested whether other carbon sources than acetate could give rise to the same correspondence of the modeling results and the experimental data. This would indicate that other metabolite exchanges, in addition to or instead of acetate, could play a role as well. Other modes of cross feeding cannot be excluded, because *E. coli* is known to excrete many other metabolites that can readily pass biological membranes, such as the weak acids occurring in central metabolism. To explore this question of alternative cross-feeding metabolites, we performed a series of simulations where we allowed the two strains to cross-feed via a set of metabolites (one-at-a-time) and minimized the glucose uptake flux to achieve the experimental growth rate with unlimited cross-feeding flux capacities. We fixed the specific growth rate at the set dilution rate and then minimized the glucose uptake rate. We then searched for single alternative cross-feeding metabolites that would give rise to the same or lower glucose uptake rate than experimentally found. In Figure 3C, the percentage change in minimum glucose flux required to achieve the same growth rate, compared to the situation when only acetate is the cross-feeding metabolite, is plotted for different alternative cross-feeding metabolites. This analysis indicates that cross feeding on other metabolites such as ethanol, pyruvate, lactate and several others cannot be excluded: they can each explain the experimental data equally well. This example illustrates the potential of cFBA in studies of microbial consortia where the cross-feeding metabolites need to be identified.

### General Modular Structure of Stoichiometric Matrices of Microbial Consortia

**Figure 4:**
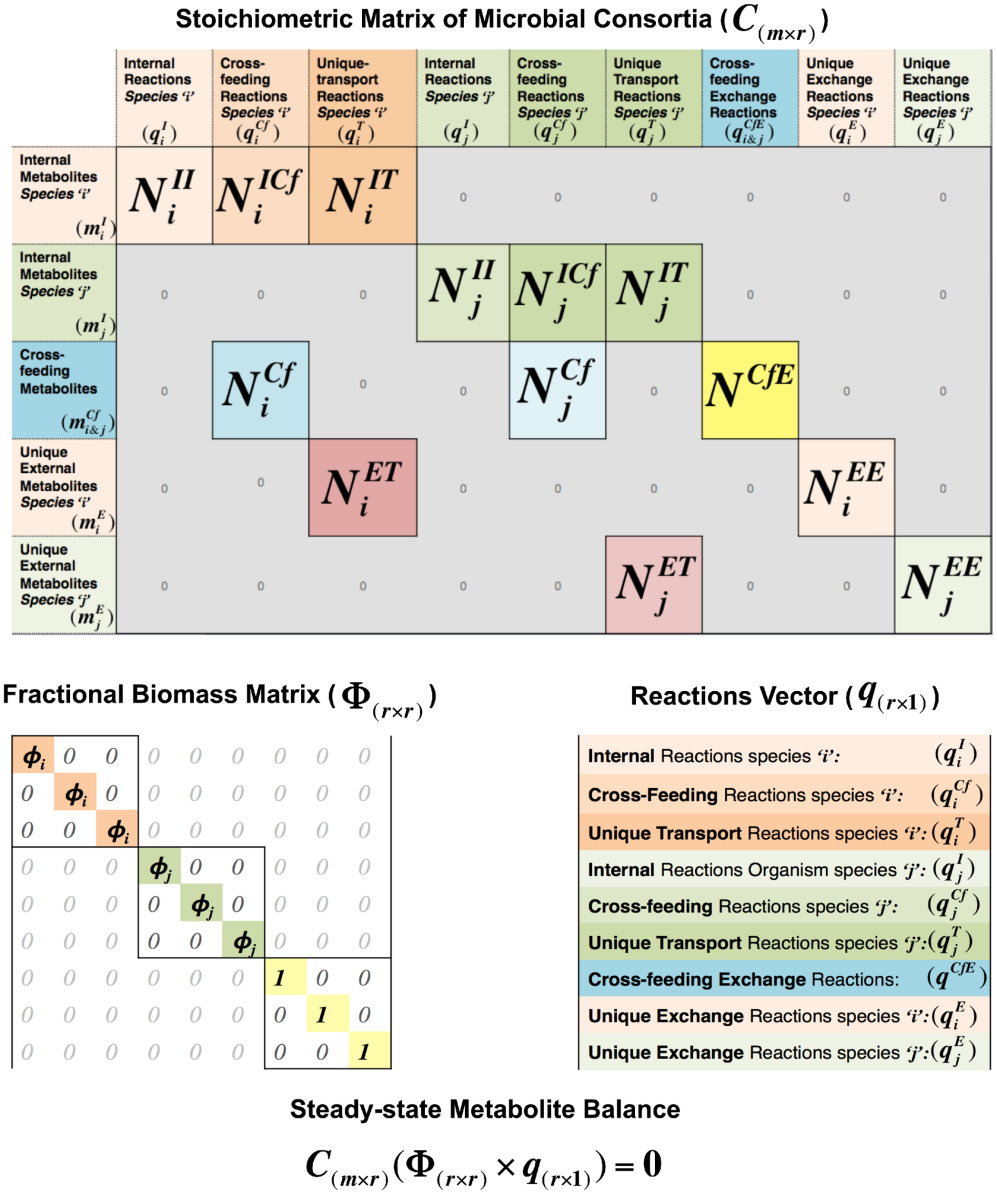
General structure of the stoichiometric matrix of a microbial consortium. The community stoichiometry matrix 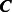 has 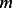 rows (metabolites) and 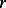 columns (reactions) and is created by merging individual stoichiometric matrices (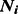 **,
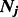
**) of community microorganisms and the environmental fluxes. The species-specific stoichiometric matrices have a consistent organization of metabolites (intracellular (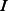), cross-feeding (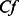) and extracellular (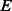)) and reactions (intracellular (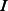), cross-feeding (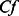), unique transport (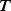) and environmental exchange (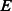)). Any sub-matrix notation has species name (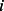 or 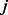) as subscript and type of metabolites and reactions as superscript. The community stoichiometry matrix multiplied by the fractional biomass matrix 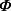 and flux vector 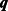 then gives the steady state mass balances of the community (equation (3)).

Equation (3) captures the entire structure of a microbial community: it considers the metabolic cross-feedings between microorganisms, the community growth rate, environmental fluxes and the fractional biomass abundances. The examples of cFBA that we have discussed so far considered two species. cFBA can straightforwardly be applied to communities with many microorganisms (in information S1). In these cases, the stoichiometric matrix of the community, denoted by 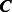, has a general modular structure. It is made up of stoichiometric matrices of participating microorganisms and its sub-matrices are functional blocks that have a clear microbial-ecological interpretation (Figure 4).

To construct the community stoichiometric matrix, the reactions of individual microorganisms involved in cross-feeding reactions and communication with the environment need to be identified. The reactions of individual species can typically be derived from genome-scale stoichiometric models that already exist but can, in principle, also be derived from reduced stoichiometric models of species that are less well characterized. Three types of metabolites and four types of reactions can be distinguished at the level of the metabolism of every microorganism. Collecting these metabolites and reactions leads to a rearranged stoichiometric matrix (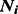) of a single microbial species 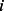 as given below.
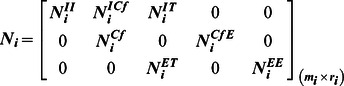



Here we distinguish the 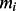 metabolites and 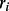 reactions that are involved in the metabolism of species 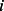. Stoichiometric coefficients for metabolites that only occur intracellularly, populate the 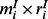 sub-matrix 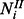. Sub-matrix 
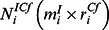
 and 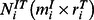 represents the stoichiometry of cross-feeding reactions (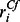) and unique transport reactions (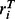) respectively, acting upon intracellular metabolites. All the extracellular metabolites can be distinguished by their roles in the consortium; some metabolites (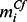) are cross-fed upon or competed for, while others are uniquely consumed or produced by specific organisms (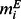). Cross-feeding reactions involved in the transport of cross-feeding or competing metabolites make up sub-matrix 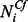 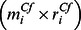 and unique extracellular metabolites taken up by unique transport reactions make up sub-matrix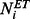 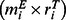. Finally, environmental exchange reactions that exchange extracellular metabolites with the environment can be classified into two subgroups depending upon the kind of extracellular metabolites they exchange in the consortium. Stoichiometric coefficients of exchange reactions transferring cross-feeding or competing metabolites to and from the environment make sub-matrix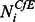 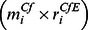, while the rest of the exchange reactions and organism specific extracellular metabolites create the sub-matrix 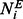 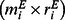. All the individual stoichiometric matrices of participating organisms can be re-arranged in these generic sub-matrices and then merged to form the matrix 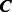 as shown in Figure 4.

## Discussion

In this paper, we presented an approach to interrelate genotype, phenotype, and community structure for microbial communities at steady state. Our cFBA method allows for the prediction of metabolic fluxes, community growth rate, and the fractional biomass abundance given (genome-scale) stoichiometric models of the participating species and constraints derived from biochemistry, thermodynamics, microbial physiology, and ecology. We derived the cFBA method from the microbiological principles of balanced growth and mass flow in microbial communities. We thus extended the concept of balanced growth of a single organism in microbiology to microbial ecology.

At present, cFBA is limited to microbial communities at balanced growth, such that all microorganisms grow equally fast and have an intracellular metabolism operating at steady state. The resulting condition of equal specific growth rate can be directly used as the objective function that is to be maximized computationally. The optimization leads to the identification of an optimal community structure. This structure encompasses the rates of all the metabolic fluxes in the community and fractional biomass abundances. cFBA applies to microbial communities where the environmental changes are slow enough for the entire community to settle to a steady state. Communities involved in wastewater treatment or bioremediation can attain steady state levels when done in specific bioreactors [32], [33], [34]. But also communities in natural environments can be exposed to fairly constant environmental conditions, such as communities found in the (deep) subsurface or on inert surfaces [23]. Another application of cFBA is the study of mixed cultures in controlled bioreactors for new environmental biotechnological applications, such as the production of bioelectricity [35] or bio-plastics [36].

Constraint-based stoichiometric modeling approaches for the metabolic networks of microbial communities, such as cFBA or other methods [11], [12], [13], [14], [15], [16], [18], [19], [20], can be a great tool to supplement experimental microbial community analysis. These computational methods can address specific questions unanswered by molecular characterization of communities and the mathematical models are natural ways to integrate heterogeneous data. For instance, after identification of the microbial species (or ecotypes) making up the community and the (partial) functional annotation of their genome, the metabolic network can be reconstructed from this genome information [8]. Then, depending on the level of genome annotation, the majority of the metabolic capacities of the microorganisms are known. How those metabolic capacities together give rise to ecosystem level properties regarding biomass abundances, growth rate, and metabolic activities can subsequently be addressed with constrained-based stoichiometric modeling approaches. The output of those computational methods can be directly compared to available experimental data about fluxes and biomass abundances. cFBA extends the growing arsenal of such computational methods and has major advantage compared to previous methods that it is straightforward and predicts the entire state of a microbial community, including biomass abundances of individual species. It can address an unlimited number of species and any type of species interaction. Future extensions of cFBA will consider dynamic scenario’s where one or several nutrients are limiting consortium growth and depleting slowly.

The constraint-based stoichiometric modeling of microbial communities is still largely in its infancy. Much is still to be learnt from studies where the predictions of these modeling approaches are critically compared to experimental data. How can we study microbial communities in a sensible fashion by using an optimization approach? Do we need to consider multi-objective nonlinear optimization approaches? If so, the computational approaches will quickly run into problems related to computational speed and uniqueness of solutions. However, even if not all the assumptions hold, as is also often the case for single-species FBA, these models will be useful to explore the metabolic potential of microbial communities. We thus expect approaches such as cFBA to be already sufficiently informative to become a vital component of the workflow of studying communities with modern “omics” approaches.

## Materials and Methods

This cFBA framework has been coded using Python™ programming language and requires CBMPy (http://cbmpy.sourceforge.net/) package and IBM ILOG CPLEX Optimizer by IBM. CBMPy has been developed based on the principles of Python Simulator of Cellular Systems (PySCes) [37]. In supplementary materials; scripts for phase plane analyses shown in Figure 2B and 2C coded in Wolfram *Mathematica®* 8, python scripts and data for analyses depicted in Figures 2A, 3B and 3C and all the stoichiometric models (SBML format) used in this paper are provided.

## Supporting Information

Figure S1 **Illustration of the reconstruction of a stoichiometric model of the metabolism of a microbial consortium.** To emphasize the steps involved in the construction of a stoichiometric model of a microbial consortium, we use reduced stoichiometric descriptions of microbial growth and product formation. The metabolic network of the first organism, species *i*, is shown in Figure S1-A and for species *j* in Figure S1-D. Three types of reactions occur in these network diagrams: intracellular (colored arrows), membrane transport (solid-black arrows), and environment exchange reactions (dashed-black arrows). Every reaction runs at a certain rate or biomass-specific flux, denoted by *q* with a unique subscript referring either to the process (anabolism *‘ana’*, catabolism *‘cat’*, respiration *‘res’* and product formation *‘pro’*) or extra-cellular metabolite names, followed by species name (*i* or *j* ) separated by underscore. And, metabolites are classified on the basis of the compartments they exist in i.e. intracellular (denoted with species name as subscript), extracellular (subscript *‘ext’*) and fixed environmental (underlined) metabolites. These specific fluxes have as their unit: mass flow per gram biomass, i.e. *mol•g^−1^•h^−1^*; and every reaction considered in these models should be elementally and charge balanced. All reactions can conveniently be expressed in terms of a stoichiometric matrix for each organism, denoted by *N* and the species name as subscript, as shown in Figure S1-B and S1-C. In Figure S1-E, the metabolic network diagram of the entire consortium is shown. Some of the products (colored boxes; succinate and ammonia) that were excreted into the environment in Figure 1A and 1D have now become cross-feeding metabolites between two species; and every extracellular metabolite can, in principle, overflow into the environment via an exchange reaction (dashed-black arrows). In the consortium, we have to consider the biomass amounts of the two species explicitly. Species-specific membrane transport fluxes should be multiplied by the abundance of the species, denoted by *x_i_* and *x_j_*.
(TIF)

Information S1 **Detailed mathematical descriptions of the optimization problem, two-species community and structure of the** ***C*** **matrix of a three-species microbial consortium.**
(PDF)

Scripts S2 **Programming scripts for reproducing data shown in** **Figure 2** **of the manuscript.**
(ZIP)

Scripts S3 **Programming scripts for reproducing data shown in** **Figure 3** **of the manuscript.**
(ZIP)
