# Supplementary figures and images for: Community Flux Balance Analysis for Microbial Consortia at Balanced Growth
Source: PLoS One. 2013 May 31;8(5):e64567. doi: 10.1371/journal.pone.0064567 (PMC3669319; doi:10.1371/journal.pone.0064567)

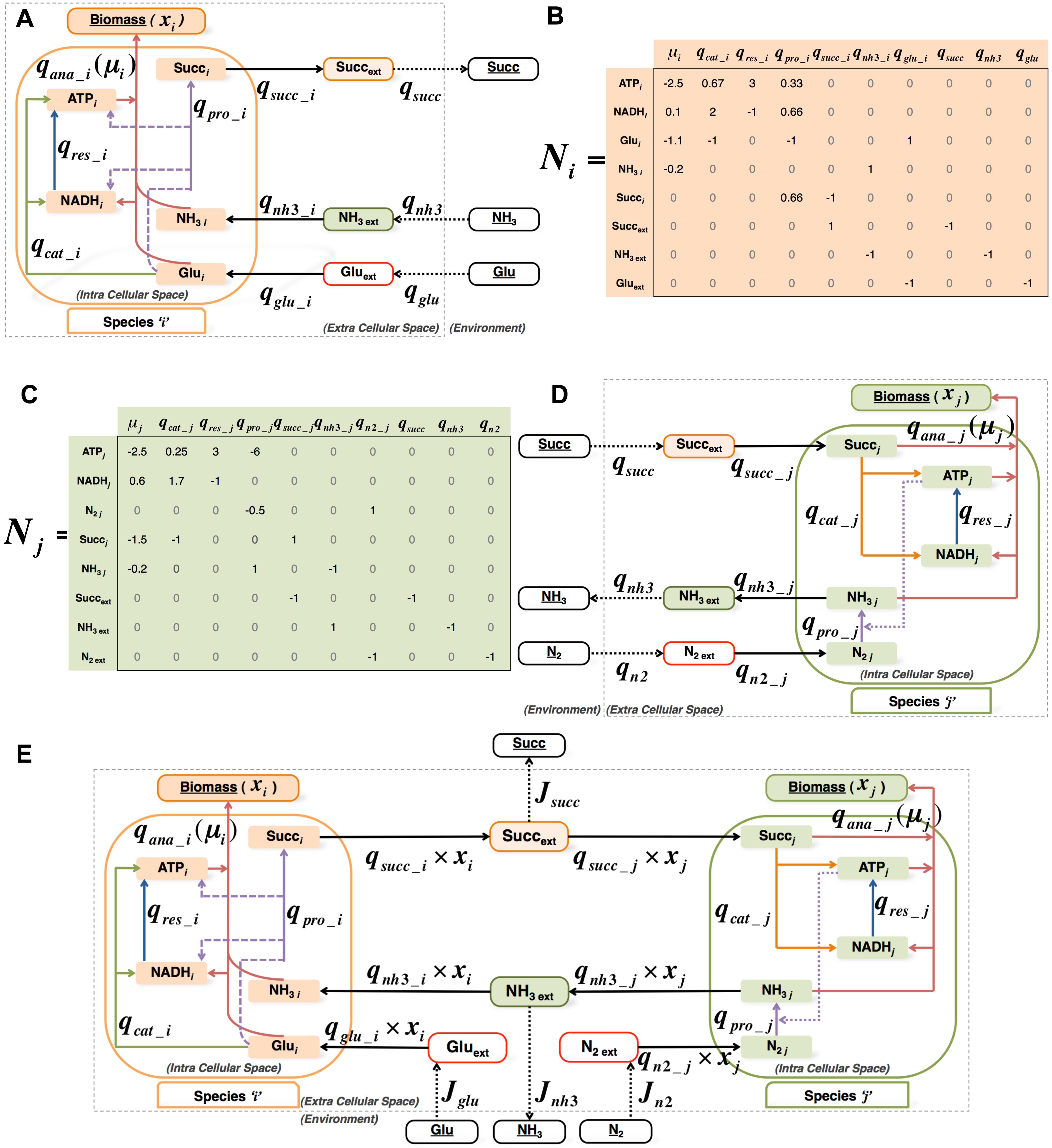

Supplement: Figure S1 — Illustration of the reconstruction of a stoichiometric model of the metabolism of a microbial consortium. To emphasize the steps involved in the construction of a stoichiometric model of a microbial consortium, we use reduced stoichiometric descriptions of microbial growth and product formation. The metabolic network of the first organism, species i, is shown in Figure S1-A and for species j in Figure S1-D. Three types of reactions occur in these network diagrams: intracellular (colored arrows), membrane transport (solid-black arrows), and environment exchange reactions (dashed-black arrows). Every reaction runs at a certain rate or biomass-specific flux, denoted by q with a unique subscript referring either to the process (anabolism ‘ana’, catabolism ‘cat’, respiration ‘res’ and product formation ‘pro’) or extra-cellular metabolite names, followed by species name (i or j ) separated by underscore. And, metabolites are classified on the basis of the compartments they exist in i.e. intracellular (denoted with species name as subscript), extracellular (subscript ‘ext’) and fixed environmental (underlined) metabolites. These specific fluxes have as their unit: mass flow per gram biomass, i.e. mol•g−1•h−1; and every reaction considered in these models should be elementally and charge balanced. All reactions can conveniently be expressed in terms of a stoichiometric matrix for each organism, denoted by N and the species name as subscript, as shown in Figure S1-B and S1-C. In Figure S1-E, the metabolic network diagram of the entire consortium is shown. Some of the products (colored boxes; succinate and ammonia) that were excreted into the environment in Figure 1A and 1D have now become cross-feeding metabolites between two species; and every extracellular metabolite can, in principle, overflow into the environment via an exchange reaction (dashed-black arrows). In the consortium, we have to consider the biomass amounts of the two species explicitly. Species-specific membr [file pone.0064567.s001.tif]
