# Supplementary material for: Community Flux Balance Analysis for Microbial Consortia at Balanced Growth
Source: PLoS One. 2013 May 31;8(5):e64567. doi: 10.1371/journal.pone.0064567 (PMC3669319; doi:10.1371/journal.pone.0064567)
Supplement: Information S1 — Detailed mathematical descriptions of the optimization problem, two-species community and structure of the C matrix of a three-species microbial consortium. (PDF) [file pone.0064567.s002.pdf]

Supplementary Information – S1

Detailed mathematical description of the optimization problem

The nonlinear optimization problem for cFBA is defined over the biomass fractions, $\boldsymbol{\phi}$, and the specific metabolic fluxes, $\boldsymbol{q}$, as variables and has as constraints the steady-state conditions for all the variable metabolites and capacity bounds for specific fluxes; we call the maximum *M*,

$$M(\bar{\boldsymbol{\phi}},\bar{\boldsymbol{q}}) = \max_{\boldsymbol{\phi},\boldsymbol{q}} \left\{ \mu_C \,\middle|\, \forall i: 0 = \overbrace{\underbrace{\sum_{j=1}^{n_X} \phi_j \left(\sum_{k=1}^{n_R} n_{ik} q_{kj} + g_{ij}\mu_C \right)}_{\text{Production and consumption of metabolite i by metabolism and growth}} + \underbrace{\sum_{l=1}^{n_E} n_{il} q_{il}}_{\text{Exchange of metaboite i with the environment}}}^{\text{steady state condition for metabolite i}}, \; \underbrace{\boldsymbol{q}^- \le \boldsymbol{q} \le \boldsymbol{q}^+}_{\text{Capacity constraints on specific fluxes}} \right\}$$

This is equation 3 of the main text.

Variables, coefficients, and vectors:

- ϕ_j biomass fraction of organism *j*
- n_{ik} stoichiometric coefficient of metabolite *i* in metabolic or transport reaction *k*
- g_{ij} stoichiometric coefficient of metabolite *i* in the biomass equation for organism *j*
- n_{il} stoichiometric coefficient of metabolite *i* in environment exchange reaction *l*
- q_{kj} specific flux of reaction *k* of organism *j*
- μ_C community growth rate, the objective
- $\boldsymbol{\phi}$ the vector with fractional biomasses
- $\boldsymbol{q}$ specific flux vector
- $\boldsymbol{q}^-$ vector of lower bound values for the specific fluxes
- $\boldsymbol{q}^+$ vector of upper bound values for the specific fluxes
- $(\bar{\boldsymbol{\phi}},\bar{\boldsymbol{q}})$ is the optimal state defined as $\arg M(\bar{\boldsymbol{\phi}},\bar{\boldsymbol{q}})$

In the applications discussed in the main text, we turn the nonlinear optimization problem into a linear program by considering the biomass fraction vector $\boldsymbol{\phi}$ as a parameter and no longer as a variable; then, we obtain the linear program with $\boldsymbol{q}$ as the optimization variables,

$$M(\boldsymbol{\phi}, \overline{\boldsymbol{q}}) = \max_{\boldsymbol{q}} \left\{ \mu_C \left| \forall i: 0 = \sum_{j=1}^{n_X} \phi_j \left(\sum_{k=1}^{n_R} n_{ik} q_{kj} + g_{ij} \mu_C \right) + \sum_{l=1}^{n_E} n_{il} q_{il}, \underbrace{\boldsymbol{q}^- \leq \boldsymbol{q} \leq \boldsymbol{q}^+}_{\substack{\text{capacity constraints} \\ \text{on specific fluxes}}} \right. \right\}$$

By scanning $M(\boldsymbol{\phi}, \overline{\boldsymbol{q}})$ as function of $\boldsymbol{\phi}$ we identify the global maximum $M(\overline{\boldsymbol{\phi}}, \overline{\boldsymbol{q}})$. For systems with many organisms a nonlinear optimization algorithm would be required. In the simplest case, a steepest ascent method will do because the LP algorithms are extremely fast and $\boldsymbol{\phi}$ is then changed in the direction of a maximal increase in $M(\boldsymbol{\phi}, \overline{\boldsymbol{q}})$. Ending up at local maxima can be prevented (to some extent) by using multiple starting conditions. This approach we have successfully tested on small microbial consortia up to 5 species.

Detailed description of the two-species community with reduced stoichiometric models

Community Matrix $\boldsymbol{C}$

To construct community matrix $\boldsymbol{C}$ for the consortium depicted in Figure S1-E, we need to rearrange stoichiometric matrices ($\boldsymbol{N}_i$, $\boldsymbol{N}_j$) of every organism. Three types of metabolites and four types of reactions can be distinguished at the level of single organism metabolism. Collecting these metabolites and reactions leads to a rearranged stoichiometric matrix ($\boldsymbol{N}_i$) of a single microbial species i, as given below.

$$\boldsymbol{N}_i = \begin{bmatrix} \boldsymbol{N}_i^{II} & \boldsymbol{N}_i^{ICf} & \boldsymbol{N}_i^{IT} & 0 & 0 \\ 0 & \boldsymbol{N}_i^{Cf} & 0 & \boldsymbol{N}_i^{CfE} & 0 \\ 0 & 0 & \boldsymbol{N}_i^{ET} & 0 & \boldsymbol{N}_i^{EE} \end{bmatrix}_{(\boldsymbol{m}_i \times \boldsymbol{r}_i)}$$

Here we distinguish the m_i metabolites and r_i reactions that are involved in the metabolism of species i. Stoichiometric coefficients for metabolites that only occur intracellularly populate the $m_i^I \times r_i^I$ sub-matrix N_i^{II}. Sub-matrix $N_i^{ICf}\left(m_i^I \times r_i^{Cf}\right)$ and $N_i^{IT}\left(m_i^I \times r_i^T\right)$ represents the stoichiometry of cross-feeding reactions (r_i^{Cf}) and unique transport reactions (r_i^T) respectively, acting upon intracellular metabolites. All the extracellular metabolites can be distinguished by their roles in the consortium; some metabolites (m_i^{Cf}) are cross-fed upon or competed for, while others are uniquely consumed or produced by specific organisms (m_i^E). Cross-feeding reactions

involved in the transport of cross-feeding or competing metabolites make up sub-matrix N_i^{Cf} $\left(m_i^{Cf} \times r_i^{Cf}\right)$ and unique extracellular metabolites taken up by unique transport reactions make up sub-matrix N_i^{ET} $\left(m_i^{E} \times r_i^{T}\right)$. Finally, environmental exchange reactions that exchange extracellular metabolites with the environment can be classified into two sub groups depending upon the kind of extracellular metabolites they exchange in the consortium. Stoichiometric coefficients of exchange reactions transferring cross-feeding or competing metabolites to and from the environment make sub-matrix N_i^{CfE} $\left(m_i^{Cf} \times r_i^{CfE}\right)$ while the rest of the exchange reactions and organism specific extracellular metabolites create the sub-matrix N_i^{E} $\left(m_i^{E} \times r_i^{E}\right)$. And the same way we can rearrange stoichiometric matrix of species j ($\boldsymbol{N}_j$) as given below.

$$\boldsymbol{N}_j = \begin{bmatrix} \boldsymbol{N}_j^{II} & \boldsymbol{N}_j^{ICf} & \boldsymbol{N}_j^{IT} & 0 & 0 \\ 0 & \boldsymbol{N}_j^{Cf} & 0 & \boldsymbol{N}_j^{CfE} & 0 \\ 0 & 0 & \boldsymbol{N}_j^{ET} & 0 & \boldsymbol{N}_j^{EE} \end{bmatrix}_{\left(\boldsymbol{m}_j \times \boldsymbol{r}_j\right)}$$

These rearranged species-specific stoichiometric matrices can be merged into a community matrix $\boldsymbol{C}$ that has a general structure as given below.

$$\boldsymbol{C} = \begin{bmatrix} \boldsymbol{N}_i^{II} & \boldsymbol{N}_i^{ICf} & \boldsymbol{N}_i^{IT} & 0 & 0 & 0 & 0 & 0 & 0 \\ 0 & 0 & 0 & \boldsymbol{N}_j^{II} & \boldsymbol{N}_j^{ICf} & \boldsymbol{N}_j^{IT} & 0 & 0 & 0 \\ 0 & \boldsymbol{N}_i^{Cf} & 0 & 0 & \boldsymbol{N}_j^{Cf} & 0 & \boldsymbol{N}^{CfE} & 0 & 0 \\ 0 & 0 & \boldsymbol{N}_i^{ET} & 0 & 0 & 0 & 0 & \boldsymbol{N}_i^{EE} & 0 \\ 0 & 0 & 0 & 0 & 0 & \boldsymbol{N}_j^{ET} & 0 & 0 & \boldsymbol{N}_j^{EE} \end{bmatrix}$$

In this $\boldsymbol{C}$ matrix, sub-matrix $\boldsymbol{N}^{CfE} = \boldsymbol{N}_i^{CfE} = \boldsymbol{N}_j^{CfE}$ because stoichiometry formed by environmental exchange reactions of cross-feeding metabolites are identical in every organism of a consortium and only one set of stoichiometry can exist after being merged.

As explained in the equation 2 of the main article, every species-specific reaction has to be multiplied by species-specific biomass fraction ($F_j = \dfrac{X_j}{\sum_{j=1}^{n_X} X_j}$, where n_X is total number of

species, X_j is gram biomass amount species j) to form mass balance equations for each metabolites. For this community of species i and j, this can be achieved by multiplying a diagonal biomass fraction matrix F and reactions vector $\boldsymbol{q}$, as given below.

$$\mathrm{F}_{(r\times r)}=\begin{bmatrix} f_i & 0 & 0 & 0 & 0 & 0 & 0 & 0 & 0 \\ 0 & f_i & 0 & 0 & 0 & 0 & 0 & 0 & 0 \\ 0 & 0 & f_i & 0 & 0 & 0 & 0 & 0 & 0 \\ 0 & 0 & 0 & f_j & 0 & 0 & 0 & 0 & 0 \\ 0 & 0 & 0 & 0 & f_j & 0 & 0 & 0 & 0 \\ 0 & 0 & 0 & 0 & 0 & f_j & 0 & 0 & 0 \\ 0 & 0 & 0 & 0 & 0 & 0 & 1 & 0 & 0 \\ 0 & 0 & 0 & 0 & 0 & 0 & 0 & 1 & 0 \\ 0 & 0 & 0 & 0 & 0 & 0 & 0 & 0 & 1 \end{bmatrix} \text{ and } \boldsymbol{q}_{(r\times 1)}=\begin{bmatrix} \boldsymbol{q}_i^I \\ \boldsymbol{q}_i^{Cf} \\ \boldsymbol{q}_i^T \\ \boldsymbol{q}_j^I \\ \boldsymbol{q}_j^{Cf} \\ \boldsymbol{q}_j^T \\ \boldsymbol{q}^{CfE} \\ \boldsymbol{q}_i^E \\ \boldsymbol{q}_j^E \end{bmatrix}$$

Here species names are given as subscripts and types of fluxes (as explained previously) are given as superscripts. Finally, steady-state metabolite balance for this consortium can be given as below given matrix multiplication equation.

$$\boldsymbol{C}_{(m\times r)}\left(F_{(r\times r)}\times \boldsymbol{q}_{(r\times 1)}\right)=0$$

Linear program for cFBA of a two species community

Above given equation leads to the following linear program for the optimization of specific growth rate (biomass yield) at a steady-state, given various biomass fractions.

$$\textit{Objective}: \quad \textit{Maximize } \mu_C, \textit{ where } (\mu_i = \mu_j \equiv \mu_C)$$

Subject to,

$$(1) \quad N_i^{II}(\Phi_i^I \theta_i^I) + N_i^{ICf}(\Phi_i^{Cf} \theta_i^{Cf}) + N_i^{IT} \theta_i^{IT} = 0$$

$$(2) \quad N_j^{II}(\Phi_j^I \theta_j^I) + N_j^{ICf}(\Phi_j^{Cf} \theta_j^{Cf}) + N_j^{IT} \theta_j^{IT} = 0$$

$$(3) \quad N_i^{Cf}(\Phi_i^{Cf} \theta_i^{Cf}) + N_j^{Cf}(\Phi_j^{Cf} \theta_j^{Cf}) + N^{CfE} \theta^{CfE} = 0$$

$$(4) \quad N_i^{ET}(\Phi_i^T \theta_i^T) + N_i^{EE} \theta_i^E = 0$$

$$(5) \quad N_j^{ET}(\Phi_j^T \theta_j^T) + N_j^{EE} \theta_j^E = 0$$

$$(6) \quad \theta^{\min} \le \theta_i^I, \theta_i^{Cf}, \theta_i^T, \theta_i^E, \theta_j^I, \theta_j^{Cf}, \theta_j^T, \theta_j^E, \theta^{CfE} \le \theta^{\max}$$

$$(7) \quad \mu_i = \mu_j$$

Note that $\mu_i \in \boldsymbol{\theta}_i^I$ and $\mu_j \in \boldsymbol{\theta}_j^I$ and notations for reactions sub-vectors ($\boldsymbol{\theta}_{species}^{reaction\ type}$) and fractional biomass sub-matrices ($\Phi_{species}^{dimension}$) are represented in a way that subscript represents species-name and superscript represents the type of reactions it contains or the dimensions (for fractional biomass sub-matrices). The objective is maximization of specific growth rate of the consortium. Both of the species are forced to have the same growth rate to make sure that the steady state condition holds during the period of balanced growth (constraint 7). Constraints 1 and 2 are the steady state conditions for the internal metabolites of species i and j. Constraint 3 represents the steady state conditions for all the cross-feeding metabolites and constraints 4 and 5 are steady state balances of the organism specific extracellular metabolites. Constraint 6 takes into account measured flux bounds and thermodynamic reversibility conditions on fluxes by indicating maximum and minimum values that a flux can obtain in the solution.

Structure of the $\boldsymbol{C}$ matrix of a three-species microbial consortium

For a three-species consortium consisting of species i, j and k, we have their individual stoichiometric matrices $\boldsymbol{N}_i, \boldsymbol{N}_j$ and $\boldsymbol{N}_k$, respectively. As we rearranged individual stoichiometric matrix for a two-species community, we can rearrange $\boldsymbol{N}_i$ in a below given sub-matrix form.

$$N_i = \begin{bmatrix} \boldsymbol{N}_i^{II} & \boldsymbol{N}_i^{ICfj} & \boldsymbol{N}_i^{ICfk} & \boldsymbol{N}_i^{ICf} & \boldsymbol{N}_i^{IT} & 0 & 0 & 0 & 0 \\ 0 & \boldsymbol{N}_i^{Cfj} & 0 & 0 & 0 & \boldsymbol{N}_i^{CfjE} & 0 & 0 & 0 \\ 0 & 0 & \boldsymbol{N}_i^{Cfk} & 0 & 0 & 0 & \boldsymbol{N}_i^{CfkE} & 0 & 0 \\ 0 & 0 & 0 & \boldsymbol{N}_i^{Cf} & 0 & 0 & 0 & \boldsymbol{N}_i^{CfE} & 0 \\ 0 & 0 & 0 & 0 & \boldsymbol{N}_i^{ET} & 0 & 0 & 0 & \boldsymbol{N}_i^{EE} \end{bmatrix}_{(m_i \times r_i)}$$

Here we distinguish the m_i metabolites and r_i reactions that are involved in the metabolism of species i in the same way as we did for the two-species consortium, previously. The only difference is in the classification of cross-feeding metabolites and reactions into pair-wise-common (between species i and j, species i and k) and common (between species i, j and k). $N_i^{ICfj}\left(m_i^I \times r_i^{Cfj}\right)$ and $N_i^{ICfk}\left(m_i^I \times r_i^{Cfk}\right)$ represents the stoichiometry of pair-wise-common cross-feeding reactions between species i and j (r_i^{Cfj}), species i and k (r_i^{Cfk}) respectively, acting upon intracellular metabolites (m_i^I). And, $N_i^{ICf}\left(m_i^I \times r_i^{Cf}\right)$ represents common cross-feeding reactions amongst all three species, acting upon intracellular metabolites. The same way, extracellular metabolites that are being cross-fed upon can be classified and matrices $N_i^{Cfj}\left(m_i^{Cfj} \times r_i^{Cfj}\right)$, $N_i^{Cfk}\left(m_i^{Cfk} \times r_i^{Cfk}\right)$ and $N_i^{Cf}\left(m_i^{Cf} \times r_i^{Cf}\right)$ represent common cross-feeding reactions and metabolites of species i that are same as species j, species k and common amongst all three species, respectively. Finally, $N_i^{CfjE}\left(m_i^{Cfj} \times r_i^{Ej}\right)$ and $N_i^{CfkE}\left(m_i^{Cfk} \times r_i^{Ek}\right)$ represents stoichiometric matrices consisting of pair-wise-common extracellular cross-feeding metabolites and their environmental exchange reactions and $N_i^{CfE}\left(m_i^{Cf} \times r_i^{E}\right)$ consists of extracellular metabolites that can be cross-fed by all three species and their environmental exchange reactions. After rearranging all three individual stoichiometric matrices, they can be merged to form the matrix $\boldsymbol{C}$ for this three species community, as shown below:

$$
C=\begin{bmatrix}
N_i^{II} & N_i^{ICfj} & N_i^{ICfk} & N_i^{ICf} & N_i^{IT} & 0 & 0 & 0 & 0 & 0 & 0 & 0 & 0 & 0 & 0 & 0 & 0 & 0 & 0 & 0 & 0 & 0 \\
0 & 0 & 0 & 0 & 0 & N_j^{II} & N_j^{ICfi} & N_j^{ICfk} & N_j^{ICf} & N_j^{IT} & 0 & 0 & 0 & 0 & 0 & 0 & 0 & 0 & 0 & 0 & 0 & 0 \\
0 & 0 & 0 & 0 & 0 & 0 & 0 & 0 & 0 & 0 & N_k^{II} & N_k^{ICfi} & N_k^{ICfj} & N_k^{ICf} & N_k^{IT} & 0 & 0 & 0 & 0 & 0 & 0 & 0 \\
0 & N_i^{Cfj} & 0 & 0 & 0 & 0 & N_j^{Cfi} & 0 & 0 & 0 & 0 & 0 & 0 & 0 & 0 & N^{CfijE} & 0 & 0 & 0 & 0 & 0 & 0 \\
0 & 0 & N_i^{Cfk} & 0 & 0 & 0 & 0 & 0 & 0 & 0 & 0 & N_k^{Cfi} & 0 & 0 & 0 & 0 & N^{CfikE} & 0 & 0 & 0 & 0 & 0 \\
0 & 0 & 0 & 0 & 0 & 0 & 0 & N_j^{Cfk} & 0 & 0 & 0 & 0 & N_k^{Cfj} & 0 & 0 & 0 & 0 & N^{CfjkE} & 0 & 0 & 0 & 0 \\
0 & 0 & 0 & N_i^{Cf} & 0 & 0 & 0 & 0 & N_j^{Cf} & 0 & 0 & 0 & 0 & N_k^{Cf} & 0 & 0 & 0 & 0 & N^{CfE} & 0 & 0 & 0 \\
0 & 0 & 0 & 0 & N_i^{ET} & 0 & 0 & 0 & 0 & 0 & 0 & 0 & 0 & 0 & 0 & 0 & 0 & 0 & 0 & N_i^{EE} & 0 & 0 \\
0 & 0 & 0 & 0 & 0 & 0 & 0 & 0 & 0 & N_j^{ET} & 0 & 0 & 0 & 0 & 0 & 0 & 0 & 0 & 0 & 0 & N_j^{EE} & 0 \\
0 & 0 & 0 & 0 & 0 & 0 & 0 & 0 & 0 & 0 & 0 & 0 & 0 & 0 & N_k^{ET} & 0 & 0 & 0 & 0 & 0 & 0 & N_k^{EE}
\end{bmatrix}
$$

This merging is done in a way that rows representing common extracellular metabolites and columns representing common environmental exchange reactions don't get duplicated. This duplication can be avoided by using matrices $N_i^{CfjE} = N_j^{CfiE} \circ \boldsymbol{N}^{CfijE}$, $N_i^{CfkE} = N_k^{CfiE} \circ \boldsymbol{N}^{CfikE}$, $N_j^{CfkE} = N_k^{CfjE} \circ \boldsymbol{N}^{CfjkE}$ and $N_i^{CfE} = N_j^{CfE} = N_k^{CfE} \circ \boldsymbol{N}^{CfE}$. This is the general structure of proposed $\boldsymbol{C}$ matrix and all the analyses explained in this paper can be performed for a community made up of any number of species.
